# The new EU General Data Protection Regulation: what the radiologist should know

**DOI:** 10.1007/s13244-017-0552-7

**Published:** 2017-04-24

**Authors:** European Society of Radiology (ESR)

**Affiliations:** Neutorgasse 9/2, 1010 Vienna, Austria

**Keywords:** Data protection, Health data, Patient rights, Research, EU regulation

## Abstract

**Abstract:**

The European Society of Radiology (ESR) informs its membership and its associated Institutional Members about the new General Data Protection Regulation (GDPR) of the European Union (EU,) which will apply from 25 May 2018. Radiologists and radiology departments should be prepared to comply with several new rules for the protection of imaging data. Although the new GDPR applies to all domains of the public and private sectors, some specific derogations are defined for data concerning health, aiming at protecting the rights of data subjects and confidentiality of their personal health data, whilst preserving the benefits of processing data, including digital images for research and public health purposes. Specific new obligations which healthcare providers (including radiologists/radiology departments) should prepare for include data access for patients, rules for data processing including explicit consent of the data subject in the absence of derogations, or technical and organisational safeguards. National health authorities can define exceptions and derogations from certain obligations by means of national law. They will also define sanctions in the form of penalties or fines that may be applicable for organisations of the public and private sector that fail to comply with the rules of the GDPR.

**Main messages:**

• *Explicit consent prior to data processing will be necessary*.

• *Explicit consent prior to communication of imaging data will be necessary*.

• *Providing patient access to their personal data*, *including portability*, *will be required*.

• *Certain derogations and exceptions exist for healthcare and research*.

• *Additional specific rules may be defined by national law*.

## Introduction

The revised General Data Protection Regulation (GDPR) of the European Union (EU) addresses the protection of its residents (data subjects) with regard to accessing, processing and the free movement of their personal data [1]. It was adopted by the European Parliament in April 2016 and shall apply across the EU from 25 May 2018. As opposed to a directive, a regulation is directly applicable in all member states. The main purpose of the GDPR is to define and update several basic rights of data subjects regarding control of and access to their personal data, and to implement common rules for data protection in all member states. At the same time, the regulation intends to enhance economic development through clear and common rules throughout the EU for all companies with business in the EU.

Key elements of the new regulation include, for example: the need for clear and affirmative consent by the data subject concerned, destruction of data if storage is no longer necessary for the initial purpose or after withdrawal of consent by the data subject (‘the right to be forgotten’); the right to obtain rectification of his/her personal data; the right of the data subject to transfer personal data to another service provider (‘data portability’); the right of the data subject to be informed when his/her data have been hacked. The new GDPR implies that all organisations processing personal data must be able to prove that they comply with the rules. It also proposes a European Data Protection Board and requires institutions which process certain types or volumes of data to have a designated data protection officer (DPO), who is in contact with the national data protection authorities. The legislation also specifies that ‘effective, proportionate and dissuasive’ penalties or financial fines may apply in the case of non-compliance with the data protection rules.

Protection of personal data is of particular importance in the health sector, and the basic requirement of confidentiality of diagnostic and therapeutic information requires special attention in the digital environment. The often conflicting objectives of ensuring privacy rights for personal data whilst providing adequate access to such data, e.g. for the vital interests of the data subject or other persons, but also for research and public healthcare purposes, represent particular challenges in this sensitive area. Therefore, the new GDPR provides several derogations with regard to data concerning health.

A comprehensive, detailed interpretation of the new GDPR is beyond the scope of the present publication. Several organisations such as the NHS European Office, the Wellcome Trust and the Westin Research Center have provided in-depth analyses [2–4], and guidelines based on the GDPR are currently being formulated by working groups with regard to specific topics and frequently asked questions, e.g. about data portability, data protection officers, and other specific topics [5]. The GDPR is not specifically designed for health data, and interpretations for certain applications may differ. Associations and other bodies representing categories of controllers or processors are encouraged to prepare codes of conduct, or amend or extend such codes, for the purpose of specifying the application of the GDPR (Article 40/2). The purpose of this publication is to summarise some important elements of the protection of digital data concerning health in the framework of the new GDPR, and to make radiologists aware of some key provisions of the new GDPR that should be taken into account in daily practice, clinical research and public health projects.

## The new GDPR and data concerning health

*Data concerning health* are defined as personal data related to the physical or mental health of a natural person (i.e. an individual), including the provision of health and care services, which reveal information about his or her health status. The term *biometric data* means any personal data resulting from specific technical processing relating to the physical, physiological or behavioural characteristics, which allow or confirm the unique identification, such as facial images or dactyloscopic data. *Genetic data* are all personal data relating to the inherited or acquired genetic characteristics of an individual, which give unique information about their physiology or health, resulting in particular from an analysis of a biological sample (Article 4).

Although the implementation of electronic patient records (EPR) and picture archiving and communication systems (PACS) still varies considerably among the different European countries, there is no doubt that the possibility of sharing digital image information offers many advantages in the domains of personal and public health, teaching and research. At the same time, recent rapid technological developments in data processing, data sharing across national European boundaries and data use for various purposes in the public and private sectors may lead to legal uncertainties with regard to privacy rights, ownership of data and data processing. Protection of data concerning health, including digital medical images, has thus become a major concern.

The new GDPR aims at protecting the rights of the data subject and confidentiality of personal health data as an important civil right, whilst preserving the benefits of digital image processing for research and public health purposes. Hospitals and other healthcare organisations should be prepared to comply with the requirements of the new GDPR. Besides the protection of confidentiality, the rights of the data subject include obtaining access to his/her personal data stored in the EPR and PACS, obtaining a copy for transferring them to another healthcare provider, and to have inaccuracies in his/her data rectified. There is also a requirement for healthcare providers to demonstrate that their procedures fulfil the conditions of lawful data processing required by the GDPR, e.g. on the basis of explicit consent of the data subject, or the use of safeguards when processing data for research or public health purposes.

The new GDPR is welcomed by the European Society of Radiology as well as numerous other health stakeholders, including the European Alliance for Personalised Medicine (EAPM), the European Organisation for Research and Treatment of Cancer (EORTC), the European Patients’ Forum (EPF) and the European Society for Medical Oncology (ESMO) [6–9].

## GDPR versus national and local rules

Close collaboration between healthcare providers and organisations and the national health authorities is recommended, because the framework provided by the GDPR enables individual member states to define rules for specific situations in national law.

Although the GDPR applies in the entire EU, it recognises the relevance of national rules and provides a certain margin of manoeuvrability for all member states to specify their rules within the general framework of the EU legislation with regard to health data. For example, it provides the possibility of derogating from the prohibition on processing sensitive categories of data if this is done by law, and subject to suitable safeguards so as to protect personal data and other fundamental rights. Member states may also introduce further conditions, including limitations, with regard to the processing of genetic data, biometric data or health data.

National supervisory authorities should provide hospitals and other health-related organisations with sector-specific advice and guidance about what will be necessary for them to demonstrate compliance with the new GDPR. For example, both public and private hospitals and other health and care providers will need to define their lawful basis for the processing of health data and demonstrate their compliance with relevant rules. This may be affected by means of explicit (documented) consent of the data subject, protection of a vital interest of the data subject or another individual, performance of a task in the interest of public health, in the context of scientific and historical research, or by statistical purposes (subject to Article 89 and national or EU law). The simple statement of ‘legitimate interest’ is no longer acceptable to justify processing of data [2]. The new GDPR provides that member states shall define ‘effective, proportionate and dissuasive’ penalties or fines that may be applicable to organisations of the public and private sectors failing to comply with the rules for data protection.

Access to databases such as EPR and PACS is currently mainly regulated by local rules, e.g. the hospital administration, or by a national authority. Access privileges in hospitals may, for example, be restricted to physicians and other health personnel in charge of an individual patient. In the interest of individual patient care, the European Society of Radiology endorses the view that the radiologist who interprets images of a patient for diagnostic purposes or who performs interventional image-based procedures should have full access privileges as a consultant to all medical data including all previous images as well as clinical, chemical and biological analyses. Access privileges to the EPR for scientific or any other use should, however, follow a clearly defined authorisation process approved by a local ethics committee and should require informed consent of the data subject with regard to a clearly defined purpose.

Since the era of paper and film, the obligations to keep records archived and accessible for a defined period of time have usually been defined, according to medical or medico-legal purposes, by national or local authorities. Over the past few years, storage of large digital data such as images on PACS has been greatly facilitated by the price decay of storage capacity. The adequate storage period for digital data on PACS and EPR depends first of all on the potential benefits of this information for the individual’s personal health and may be 10 years or longer, e.g. in the context of chronic conditions originating before or during childhood, or neoplastic conditions with a propensity for late recurrence. As long as confidentiality and data safety are guaranteed and access rules are defined with regard to the purpose of individual healthcare, there appears to be no obvious reason and no requirement to erase data such as medical images routinely after defined periods without the data subject’s request. Interpretations may differ with regard to derogations from the data subject’s right to erasure upon his/her request in the context of health data (Articles 9, 17 and 89) [2, 3]. Specific requirements in this context may still have to be clarified by national legislation of member states [3].

## Portability of health data

The data subject will have the right to obtain any personal data that are processed by automated means. Unless defined otherwise by national law, hospitals and other healthcare providers, such as radiology departments, may have to be prepared to provide electronic data in an appropriate format to a patient upon request, so he or she can choose to consult with another provider of care, regardless of national borders. Such copies of data records have to be provided free of charge. Charges can only be made for further copies or where requests for information are ‘manifestly unfounded or excessive’. Further detail on data portability is provided by a recent publication from the EU working group [5].

## Personal data breach

Personal data breach means a breach of security leading to the accidental or unlawful destruction, loss, alteration, unauthorised disclosure of, or access to, personal data transmitted, stored or otherwise processed. In addition to the obligations to communicate a personal data breach to the data subject, data breaches must be notified within 72 h to the supervisory authority. Larger institutions as defined in the legislation should designate a data protection officer. Where breaches are not notified in situations where derogations exist, records still need to be kept. Encryption (see below) avoids breach notification, provided that it has been competently implemented from a technical and organisational point of view.

## Anonymisation, pseudonymisation and encryption

In order to facilitate the use of data in the context of research projects, public health or biobanks as well as ‘big data’ analytics, while protecting personal data, the new GDPR proposes technical and organisational measures such as anonymisation, pseudonymisation and encryption. The term *anonymisation* refers to removing personally identifiable information where it is not needed (e.g. the name of the patient and institution, the date and other text data on images, and perhaps even DICOM metadata from the PACS file which would allow re-identification). *Pseudonymisation* refers to the users replacing personally identifiable material with artificial identifiers. It means the processing of personal data in such a way that the data can no longer be attributed to a specific data subject without the use of additional information, as long as such additional information is kept separately and subject to technical and organisational measures to ensure non-attribution to an identified or identifiable natural person. Recital 26 (an explanatory text which is part of the legislation that sets out reasons for the provisions of Article 4) explains the following: data which have undergone pseudonymisation, which could be attributed to a natural person by the use of additional information, should be considered as information on an identifiable natural person. To determine whether a person is identifiable, account should be taken of all the means reasonably likely to be used, such as singling out, either by the controller or by any other person, to identify the individual directly or indirectly. To ascertain whether means likely to be used to identify the individual are reasonable, account should be taken of all objective factors, such as the costs of and the amount of time required for identification, taking into consideration both available technology at the time of the processing and technological development [2].

*Encryption* refers to the encoding of messages that can only be read by authorised persons. This can be done by using anonymised or pseudonymised data.

The choice of the appropriate safeguard (e.g., anonymisation or pseudonymisation) will depend on the purpose of data processing. Following pseudonymisation, attribution of data to individuals still remains possible whereas this is in principle not the case with anonymisation.

The new GDPR intends to make ‘data protection by design and by default’ an essential principle and aims to incentivise businesses to innovate and develop new ideas, methods, technologies and tools for security and protection of personal data.

It is important to recognise, however, that these technical and organisational measures and safeguards are unlikely to guarantee absolute confidentiality in the case of image-based information. We have to recognise that in the era of digital robots that can cross-match large amounts of data, absolutely reliable protection of individual digital biometric data in general, and imaging data in particular, is almost impossible once the data have become accessible. Take the example of an MR study of the head: effective anonymisation would not only require removal of all written information from the file including DICOM metadata, but also the use of a software that can provide irreversible scrambling of the soft tissue structures of the individual’s face. Still, matching by digital robotic algorithms of any particularity of organs and pathologies seen on other studies of the same individual could possibly allow re-identification.

## Conditions of lawful data processing for research

Owing to the potential public health interest created by the scientific use of ‘big data’ from biobanks and the development of ‘personalised medicine’, certain exemptions apply to the restriction of personal data for scientific research and storage in the public interest as outlined in Article 89 of the GDPR.

The key question in the context of storage of health data is always with regard to the purpose. The GDPR provides several exemptions and derogations for the use of health data, e.g. in the context of research or public health purposes under certain conditions. Typical procedures in this context include the application of ethical standards for scientific research as mentioned in Recital 33 and the implementation of organisational and technical safeguards as mentioned in Article 89 including anonymisation, pseudonymisation and encryption [2]. Pseudonymisation is generally recommended as long as the research purposes can be fulfilled in this manner. With the condition of such safeguards, the GDPR creates exemptions for researchers to process sensitive health data even beyond the purposes for which they were collected. In addition, the GDPR provides some room for manoeuvre for national regulations (derogations defined by a law) in this context. It is clearly emphasised, however, that exemptions and derogations for research purposes should not result in personal data being processed for other purposes by third parties such as employers, insurance or banking companies (Recital 54, [1].

Discussions of the legal framework regarding research purposes have been provided by recent publications [3, 4]. Nonetheless, specific questions may still need to be addressed, e.g. with regard to the technical and organisational safeguards for data sharing and processing, when transferring image data to third countries orinternational organisations, in the context of biobanks or when using cloud processing. Codes of conduct for such applications may be elaborated and proposed by scientific associations or national data protection authorities. The Biobanking and BioMolecular resources Research Infrastructure (BBMRI-ERIC) has recently published its position with regard to frequently asked questions in the context of data protection for biobanks under the new GDPR [10].

## Summary

Radiologists and national radiological societies throughout Europe should be aware of the new standards of the GDPR which will apply from 2018. The regulation aims at protecting the confidentiality of personal health data whilst preserving the benefits of digital image processing for research and public health purposes. Radiologists and radiology departments should get prepared to comply with several new rules for the protection of digital imaging data, including:

- To obtain explicit consent from the data subject prior to processing or communication his or her data unless in situations where derogations exist.
- To apply appropriate technical and organisational safeguards such as anonymisation, pseudonymisation and encryption for data use in the context of public health projects, individual research projects, or imaging biobanks for ‘big data’ analysis.
- To provide access for the data subject (i.e. the patient) to his/her personal medical records containing information such as diagnoses, examination results, assessments by treating physicians and any treatment or interventions provided.
- To provide a digital copy of personal data free of charge, including images in a structured, commonly used and machine-readable format upon patients’ request in order to enable consultation by another healthcare provider across national boundaries (‘data portability’).
- To rectify inaccurate data.
- To notify the national supervisory authority within 72 h in case of breach of personal data (or record keeping in the case of derogation).
- To designate a data protection officer (DPO) for larger institutions or for institutions processing particular types or volumes of data. These are defined in the legislation.

Rules applying in individual member states may either be specified through the GDPR itself or through national law within the framework provided by the GDPR, or defined through the European Data Protection Board.

The GDPR also encourages associations and groups of processors or controllers to propose codes of conduct with regard to specific applications and to submit them to the competent data protection authority for validation. It is worth mentioning that the new GDPR also mentions sanctions, such as penalties and fines, to be applicable to organisations of the public and private sectors failing to comply with the rules for data protection.
